# The Traditional Chinese Medicine and Relevant Treatment for the Efficacy and Safety of Atopic Dermatitis: A Systematic Review and Meta-Analysis of Randomized Controlled Trials

**DOI:** 10.1155/2017/6026434

**Published:** 2017-06-21

**Authors:** Zhao-feng Shi, Tie-bing Song, Juan Xie, Yi-quan Yan, Yong-ping Du

**Affiliations:** ^1^Department of Traditional Chinese Medicine, Xijing Hospital Affiliated to Fourth Military Medical University, Xi'an 710032, China; ^2^Department of Orthopaedics, Xi'an City Hospital of Traditional Chinese Medicine, Xi'an 710021, China

## Abstract

**Background:**

Atopic dermatitis (AD) has become a common skin disease that requires systematic and comprehensive treatment to achieve adequate clinical control. Traditional Chinese medicines and related treatments have shown clinical effects for AD in many studies. But the systematic reviews and meta-analyses for them are lacking.

**Objective:**

The systematic review and meta-analysis based on the Preferred Reporting Items for Systematic Reviews and Meta-Analyses (PRISMA) statement were conducted to evaluate the efficacy and safety of traditional Chinese medicines and related treatments for AD treatment.

**Methods:**

Randomized controlled trials (RCTs) were searched based on standardized searching rules in eight medical databases from the inception up to December 2016 and a total of 24 articles with 1,618 patients were enrolled in this meta-analysis.

**Results:**

The results revealed that traditional Chinese medicines and related treatments did not show statistical differences in clinical effectiveness, SCORAD amelioration, and SSRI amelioration for AD treatment compared with control group. However, EASI amelioration of traditional Chinese medicines and related treatments for AD was superior to control group.

**Conclusion:**

We need to make conclusion cautiously for the efficacy and safety of traditional Chinese medicine and related treatment on AD therapy. More standard, multicenter, double-blind randomized controlled trials (RCTs) of traditional Chinese medicine and related treatment for AD were required to be conducted for more clinical evidences providing in the future.

## 1. Introduction

Atopic dermatitis (AD) is a common chronic, inflammatory, and pruritic noncontagious superficial skin disease, affecting approximately up to 25% of children and 2% to 3% of adults and making the incidence rate reach a peak compared with the past few years [1]. The situation in China is also severe: 8.3% of children aged between 3 and 6 years are wrecked by this disease [2]. AD brings much financial burden to the world countries on this condition: in the United States, for instance, the national expenditure for AD ranged from $364 million to $3.8 billion dollars [3]. The combined factor of etiological candidate genes (CTLA4, IL18, TLR9, CARD4, TGF*β*1, etc.) and environment (food, sweating, stress, temperature, etc.) is the main etiology of atopic dermatitis [4, 5]. The pathogenesis of AD is still unclear nowadays, but some studies illustrate that the Th2 cell-related cytokines, chemokines, and eotaxin might be the dominant mechanisms of AD [1, 6].

The current diagnostic criterion of AD was proposed by Hanifin and Rajka in 1980 [7]. China applies the Williams diagnostic criteria for AD where the specificity and sensibility are same as Hanifin and Rajka criteria [8, 9]. If physicians want to evaluate the severe degree of AD, SCORing Atopic Dermatitis (SCORAD), Eczema Area and Severity Index (EASI), and computerized visual analogue scale (VAS) are recommended as severity system [10, 11]. The SCORAD is the well-accepted international criterion among them, including several elements and degrees such as the area of rash, the severity of rash (erythema, exudation, lichenification, etc.) and the personal symptom feeling (pruritus, agrypnia, etc.) [12]. The AD treatment is based on the precise diagnostics by physicians and personal assessment by patients. The patients who were beset by AD should understand how to keep away from stimulating factors, such as dust mite and pollen and protect skin from dryness, injury, or infection, through reasonable measures. The first-line therapy of AD contains emollients, topical steroids, topical immunosuppressant, topical nonsteroidal anti-inflammatory drugs (NSAIDs), and oral medicine (antihistaminics, steroid, or immunosuppressant) [12–15]. The adverse effects of these methods are obvious: the topical steroid can bring folliculitis, skin atrophy, striae, erythema, and infection; burning stimulation, infection, and skin cancer can present in patients who applied immunosuppressant; patients applying the antihistaminics can suffer convulsion, day mouth, vomiting, and abnormal liver function [16, 17]. Physicians have tried many possible alternative medical ways in order to avoid the adverse effects mentioned above. The symbolic examples of alternative medicines for AD, including traditional Chinese medicine and related therapy, ultraviolet radiation, and psychological therapy, have brought benefit to patients to a certain degree.

Traditional Chinese medicine has a long applying history in China for AD therapy [18]. The relevant treatment of TCM, such as acupuncture and moxibustion, could also help AD patients to alleviate their suffering. Although amount of published randomized controlled trials and systematic reviews have evaluated the TCM and relevant treatment for AD, the efficacy and safety are still unclear and evaluating criteria are limited [19, 20]. Owning to the long history and wide spreading of TCM, many classical TCM prescriptions have changed their names and obtained diversified national characteristics.

Hwangryunhaedoktang, the prescription in Korea applying for AD therapy, actually derived from the ancient* (the Eastern Jin Dynasty)* Chinese medical book named* Zhouhoufang* [21]. Acupuncture and moxibustion have played an essential role as a complementary and alternative medicine (CAM) therapy for allergy relief (itch, asthma, and allergic rhinitis) and could be recommended for AD therapy. There were no systematic review and meta-analysis articles that evaluated the effectiveness and safety of TCM and relevant treatment as an integral part for AD therapy. So we conducted a systematic review and meta-analysis to investigate the evidence of TCM and related treatment for AD treatment.

## 2. Methods

This systematic review and meta-analysis was based on the guidelines of the Preferred Reporting Items for Systematic Reviews and Meta-Analyses (PRISMA) statement [22].

### 2.1. Search Protocol

**Figure 1:**
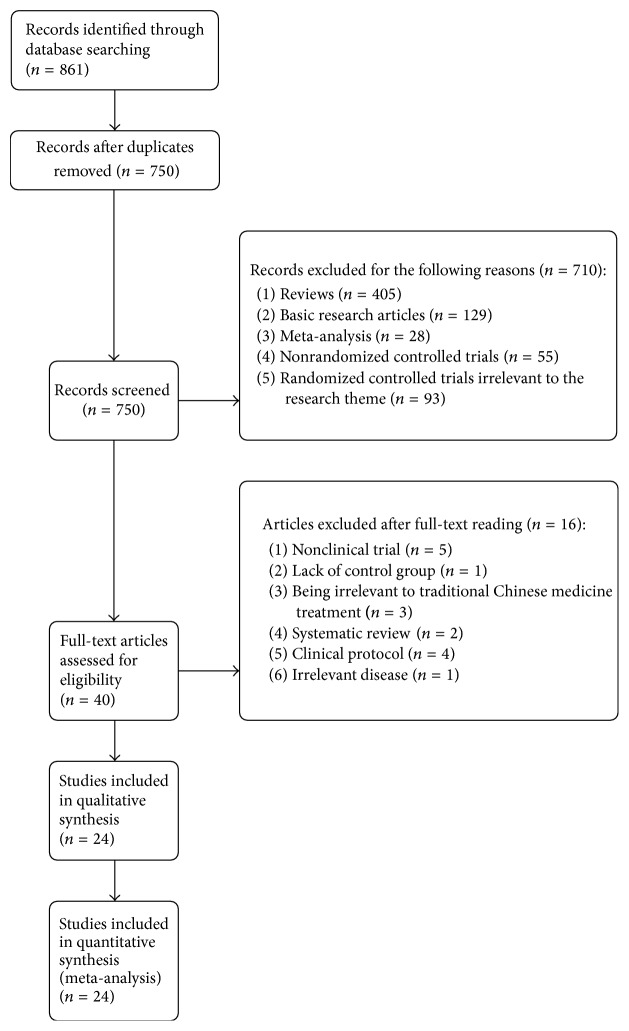
The Preferred Reporting Items for Systematic Reviews and Meta-Analyses (PRISMA) flow diagram.

Because of the pervasive low literature quality in Chinese electronic databases, so the international mainstreamed electronic databases including PubMed, Embase, Science Direct, Medline, Web of Science, ProQuest, Springerlink, and Wiley Library Online were selected for articles extraction from their inception up to December 2016 by two reviewers' (Zhao-feng Shi, Yi-quan Yan) cooperation. There were no language restrictions on literature searching in the selected database. The searching terms were conducted as follows: “allergic dermatitis”, “contact dermatitis”, “urticaria”, “drug eruption”, “eczema”, “traditional Chinese medicine”, “Chinese herbal medicine”, “Chinese medicine”, “acupuncture”, “moxibustion”, “random”, “randomized controlled trial”, and the related synonyms; meanwhile the term searching process also conducted the free text strategy and Medical Subject Headings (MeSH) term. The searching language was slightly adjusted based on the demand of different databases for adaptation. The hand searching process was also performed in the library of Fourth Military Medical University through the magazines to identify other potential related articles.  Figure 1 clearly illustrated the literature identification, inclusion, and exclusion.

### 2.2. Studies Selection

This studies selection was conducted by two reviewers (Zhao-feng Shi, Tie-bing Song) to ensure the quality of searching. The disagreement was resolved by the third reviewers (Juan Xie) or repeated discussion. Eligible literatures were included in the meta-analysis if they suited the following criteria: (1) full-text randomized controlled trials; (2) participants of any age or gender diagnosed with AD or its relevant diseases (contact dermatitis, urticaria, drug eruption, eczema, etc.); (3) no active skin exudation or infection was found before experiment; (4) experimental group: patients who received traditional herbal medicine and related preparations or formula, acupuncture and moxibustion, and any treatment having the TCM characteristics, combined or not with the western medical therapy; (5) control group: participants who received placebo or western medicines; (6) all included patients having the need to stop the previous applied topical steroid or immunosuppressant at least 1 week before RCT was conducted; (7) more than 1 severe degree criterion of AD as follows: SCORAD, EASI, VAS, simple scoring system, the Children's Dermatology Life Quality Index (CDLQI) [23], and itch intensity; (8) the major clinical outcome effectiveness of AD that was evaluated having one indicator at least as follows: (a) clinical effects; (b) the median clinical scores variation; (c) laboratory index; (d) the variation of symptom scores; (e) adverse effects; (f) lesion size change. Studies would be excluded if they do not meet the criteria mentioned above or the following: (1) severe clinical illnesses (such as liver or kidney disease) or infections; (2) absence or inconsistency of methods and results; (3) case report, comments, reviews, editorials, letters, and so forth.

### 2.3. Data Extraction and Quality Analysis

The literature data were extracted by two independent reviewers (Zhao-feng Shi, Yi-quan Yan) who deeply assessed the article inclusion and exclusion criteria based on the modern standardized procession. All discrepancies divided by two reviewers were judged by a third reviewer (Juan Xie) until they drew the final conclusion. The literature characteristics comprised the following items: (1) the name of author and the year of publication; (2) sample size (experimental group/control group); (3) participants' age; (4) participants' gender (experimental group/control group); (5) study design; (6) disease category; (7) diagnostic and evaluating criteria; (8) treating method; (9) treating duration; (10) dropout numbers; (11) major outcome parameter; (12) side effect. The authors of included articles were contacted if we could not obtain the literature characteristics mentioned before (Table 1).

Two independent investigators (Zhao-feng Shi, Tie-bing Song) conducted the study quality analysis based on the Cochrane Collaboration's tools for the risk of bias assessment [24]. This tool contained six domains for the literature's evaluation: (a) the random allocation method; (b) the allocation concealment; (c) the blinding method; (d) the outcome data integrity; (e) the outcome data of selective reports; (f) other bias sources. The study sheet was conducted which contained the evaluating criteria named “Y,” “N,” and “U” (“Y” refers to “yes,” “N” refers to “no,” and “U” refers to “unclear”). If one included study had three or more “Y,” this study could be recognized as being of high quality which has low risk of bias, and vice versa. The literature data were collected and analyzed efficiently by the software Review Manager (RevMan; version 5.2; the Nordic Cochrane Center, the Cochrane Collaboration, 2012 Copenhagen, Denmark) and presented at table (Table 2).

### 2.4. Statistical Analysis

Data of articles were pooled and analyzed by the software Review Manager (RevMan; version 5.2; the Nordic Cochrane Center, the Cochrane Collaboration, 2012 Copenhagen, Denmark). The risk ratio (RR) with 95% confidence interval (CI) for dichotomous outcomes and the Standard Mean Difference (Std. MD) or Mean Difference (MD) with 95% confidence interval (CI) for continuous outcomes was calculated, respectively, by the author (Zhao-feng Shi) and repeating verified by the author (Yi-quan Yan). The calculation of *I*^2^ statistic was applied for the statistical heterogeneity for the purpose of explanation of potential inconsistency across the included studies. It was a quantitative tool that could provide an estimate of variation resulting from heterogeneity. If the result of *I*^2^ statistic is between 25% and 50%, it would be recognized as being of low heterogeneity; the result between 50% and 75% would be of moderate heterogeneity and above 75% would be of high heterogeneity. The result of *I*^2^ statistic that exceeded 50% was regarded as obtaining the heterogeneity according to the Cochrane Handbook (version 5.1.0) notation [25]. When the heterogeneity did not exist or was moderate, a fixed-effects model applying the Mantel-Haenszel method was performed to analyze data; when the heterogeneity was high, a random effects model applying the Der Simonian-Laired method was conducted [26, 27]. The subgroup and sensitive analysis was adopted on the condition of high data heterogeneity in order to find the source of heterogeneity. The publication bias was evaluated by funnel plot, Egger's test, and Begg's test through the Stata (version 11.0, StataCorp LP, College Station, US): the asymmetry of image in visual appearance or *P* value lower than 0.05 in Egger's test or Begg's test calculation could be considered as having the publication bias [28, 29].

## 3. Results

The article flowchart for inclusion and exclusion of meta-analysis was presented on Figure 1. A total of 861 potential literatures were identified according to the search strategies from 8 electronic databases. Of these potential articles, one hundred and eleven articles were excluded for duplication and further 710 publications were excluded for the following reasons: (1) four hundred and five studies were reviews; (2) one hundred and twenty-nine studies were basic research articles; (3) twenty-eight studies were meta-analysis; (4) fifty-five studies were nonrandomized controlled trials; (5) ninety-three studies were randomized controlled trials but did not meet the article theme. Forty full-text articles were assessed for eligibility and 16 of them were excluded after full-text reading: (1) five studies were nonclinical trials; (2) one study lacked control group; (3) three studies were unrelated to traditional Chinese medicine therapy; (4) two articles were systematic reviews; (5) four studies were clinical protocols; (6) one study focused on irrelevant disease. Overall, a total of 24 articles of 1,618 patients were enrolled in this meta-analysis [30–53] (Table 1).

### 3.1. The Characteristics and Quality Evaluation of Included Articles

Table 1 clearly illustrates details and characteristics of included 24 articles. From the table, articles were grouped based on the sample size, age, gender, study design, disease category, diagnosis and evaluating criteria, treating methods, treating duration, dropout, major outcome parameters, and side effects. These articles were all strictly randomized controlled trials (RCTs) and conducted in different areas around the world. Of the final included articles, one was performed in the United Kingdom (UK) [30], one in India [36], one in Japan [40], one in Korea [46], one in Iran [52], one in the United States [47], three in Germany [41, 44, 48], and fifteen in China [31–35, 37–39, 42, 43, 45, 49–51, 53]. The year of publishing for articles was between 1992 and 2016. Overall, five hundred and seventy-eight people with AD (experimental) and 533 people without AD (control) contributed to the meta-analysis, whereas 5 articles did not show the group dividing situation (507 people) [30, 31, 33, 41, 48, 51]. The age of included people disturbed by AD had a wide range from infant to elderly people. The diagnostic and evaluating criterion of AD in different centers did not reach an agreement and kept diversified. The SCORAD [34, 43, 44, 46, 48, 50, 51] and the Hanifin and Rajka criteria [36, 42, 43, 46, 51–53] were major diagnostic and evaluating criteria of studies. Traditional herbal medicine and related preparations [30, 31, 33–37, 40, 42, 43, 46, 49, 51, 53], acupuncture and related needling methods [32, 39, 41, 44, 45, 47, 48, 50], and Chinese herbal medicine extraction [36, 52] were the applied traditional Chinese medicines and related treatments of included articles in experimental groups. Treating duration of included articles was diversified ranging from 4 days to 5 months and all articles reported the treating duration. As for side effects, thirteen articles were not reported for any reasons [32, 38–41, 44–50, 53]. Our reviewers of meta-analysis had tried to contact the authors by e-mail but did not get useful information.

**Figure 2:**
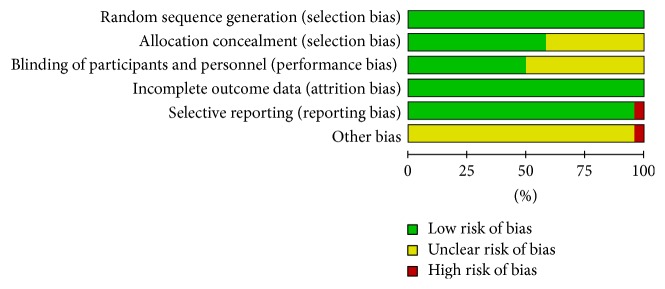
The risk of bias graph.

**Figure 3:**
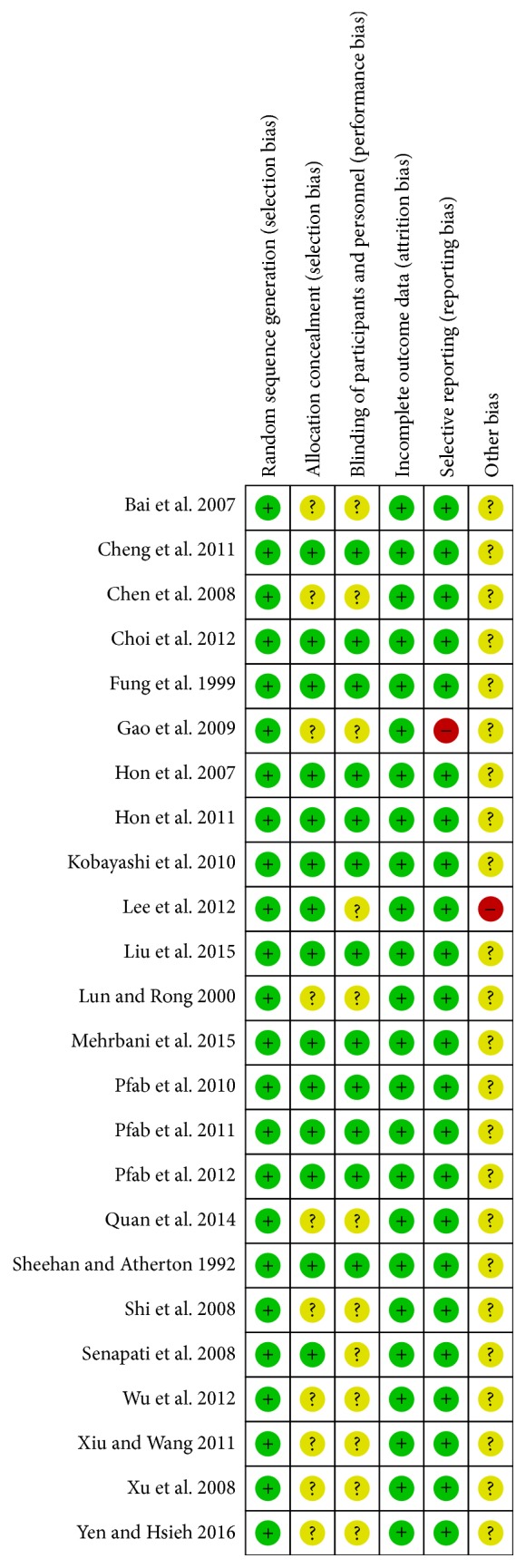
The risk of bias summary.

For all included studies in Table 2, random collection methods were perfectly presented and the major method was performed by random number table. Thirteen articles provided the allocation concealment method [30, 31, 34, 36, 40–44, 46–48, 51, 52] and eleven articles showed the blinding method [30, 31, 34, 40, 41, 43, 44, 46, 48, 51, 52]. All outcome data of included trials were integrity. Only one included article did not conform to the outcome data of selective report because the outcomes were merely presented by *P* value without specific outcome data supporting [39]. For the part of no other bias source, one article did not accord with it because there were a small number of subjects in each group, leaving the possibility that the results may have been due to chance or other bias [47]. Figures 2 and 3 were conducted by the software RevMan, both supporting the data of Table 2. Two reviewers (Zhao-feng Shi and Yi-quan Yan) conducted Tables 1 and 2 separately and disagreements of them were discussed with the third reviewer (Juan Xie) until the final same conclusion was made.

### 3.2. Meta-Analysis

#### 3.2.1. Clinical Effectiveness

**Figure 4:**
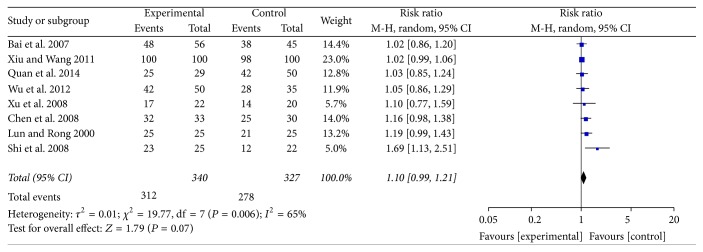
The forest plot of clinical effectiveness for traditional Chinese medicine and relevant treatment versus placebo or western medicine in AD treatment (M-H: Mantel-Haenszel estimates; CI: confidence interval).

Figure 4 showed the clinical effectiveness of traditional Chinese medicines and relevant treatments for atopic dermatitis (AD) therapy. Eight articles which were all carried out in China including 667 participants were analyzed in the forest plot of meta-analysis. For the eight included articles, five of them (62.5%) [33, 35, 37, 38, 49] performed traditional Chinese herbal medicine in the experimental group while three of them (37.5%) [32, 45, 50] performed acupuncture related therapies (acupoint injection with autoblood [32], flying needle [50], and auricular acupuncture [45]). Five studies were published before 2010 and three were published after 2010. The heterogeneity of clinical effectiveness was moderately high (*I*^2^ = 65%), so the random effects model was performed to calculate the combined data by Mantel-Haenszel test. The subgroup analysis was conducted based on the different treating methods (traditional Chinese medicine and related preparation, acupuncture, and related therapy) and publishing year of articles (before 2010, after 2010) to analyze the source of heterogeneity. Unfortunately, we still did not find the source of heterogeneity. The meta-analysis illustrated that the clinical effectiveness of AD for the experimental group which was performed by traditional Chinese medicines and relevant treatments was irrelevant to the control group (RR = 1.10, 95% CI = 0.99 to 1.21, and *P* = 0.07 > 0.05). There was no statistical difference for the result of clinical effectiveness.

#### 3.2.2. SCORAD Amelioration

**Figure 5:**
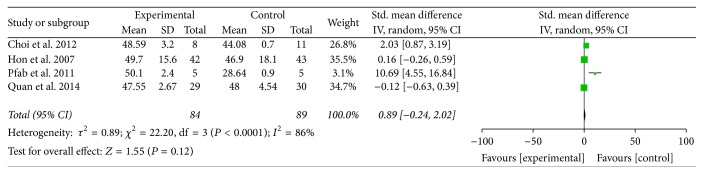
The forest plot of SCORAD amelioration for traditional Chinese medicine and relevant treatment versus placebo or western medicine in AD treatment (I-V: inverse variance; CI: confidence interval).

Figure 5 was the forest plot which illustrated the SCORing Atopic Dermatitis (SCORAD) amelioration after traditional Chinese medicines and relevant treatments for experimental groups and western medicine or placebo treatments for control groups. Four articles including 173 patients were collected and analyzed in the forest plot of meta-analysis [43, 44, 46, 50]. Standard Mean Difference (Std. MD) was conducted as a combined statistics for the cause of the obvious difference of standard deviation (SD) in the same articles' SCORAD result. Of the included studies, two (50%) conducted traditional Chinese herbal medicine as experimental method [43, 46] and others (50%) conducted acupuncture [44] and flying needle [50]. The heterogeneity was so high (*I*^2^ = 86%) that the random effects model was conducted to calculate the combined data by inverse variance (IV) approach. The subgroup analysis was performed based on the treating methods (traditional Chinese medicine and related preparation, acupuncture and related therapy) to find the source of heterogeneity. However, the result still could not provide the certain conclusion. The meta-analysis showed that the SCORAD amelioration for the experimental group and the control group was irrelevant (Std. MD = 0.89, 95% CI = −0.24 to 2.02, and *P* = 0.12 > 0.05). The result of SCORAD amelioration had no statistical difference.

#### 3.2.3. EASI Amelioration

**Figure 6:**
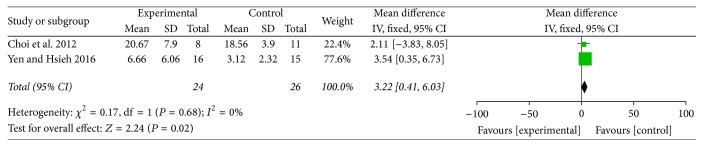
The forest plot of EASI amelioration for traditional Chinese medicine and relevant treatment versus placebo or western medicine in AD treatment (I-V: inverse variance; CI: confidence interval).

Figure 6 showed the Eczema Area and Severity Index (EASI) amelioration after traditional Chinese medicines and relevant treatments for experimental groups and western medicine or placebo treatments for control groups. Only 2 studies which incorporated 50 participants were analyzed in meta-analysis [46, 53] and Mean Difference (MD) was conducted as a combined statistics. Both the included trials chose traditional Chinese herbal medicine in the experimental group for AD therapy and were published in recent years. There was no heterogeneity (*I*^2^ = 0%) in the meta-analysis, so the fixed-effects model was conducted to calculate the combined data by inverse variance (IV) approach. The meta-analysis revealed that traditional Chinese herbal medicine was superior to western medicine for EASI amelioration in AD (Std. MD = 3.22, 95% CI = 0.41 to 6.03, and *P* = 0.02 < 0.05). The result had statistical difference.

#### 3.2.4. SSRI Amelioration

**Figure 7:**
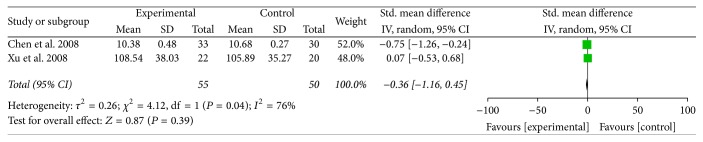
The forest plot of SSRI amelioration for traditional Chinese medicine and relevant treatment versus placebo or western medicine in AD treatment (I-V: inverse variance; CI: confidence interval).

Figure 7 illustrated the Symptom Score Reducing Index (SSRI) amelioration after traditional Chinese medicines or related treatments for experimental groups and western medicine or placebo treatments for control groups. Only 2 appropriate articles including 105 patients were collected and analyzed in meta-analysis [35, 38]. Standard Mean Difference (Std. MD) was conducted as a combined statistics for the cause of the obvious difference of standard deviation (SD) of the same article in SSRI result. Two included articles both used traditional Chinese herbal medicines in the experimental group for AD treatment. We found that the heterogeneity of included studies was obvious (*I*^2^ = 76%) maybe because of the variation of Chinese medicine ingredients between two trials and measurement time. The random effects model was conducted to calculate the combined data by inverse variance (IV) approach. The meta-analysis showed that the SSRI amelioration for the experimental group and the control group was irrelevant (Std. MD = −0.36, 95% CI = −1.16 to 0.45, and *P* = 0.39 > 0.05). The result of SSRI amelioration had no statistical difference.

#### 3.2.5. Funnel Plot Characteristics

**Figure 8:**
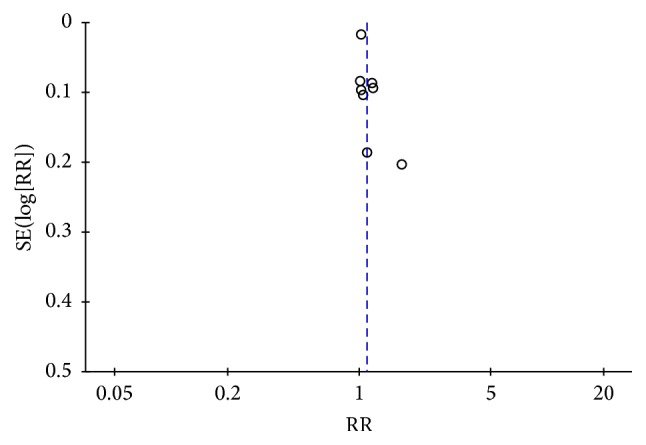
The funnel plot of clinical effectiveness.

**Figure 9:**
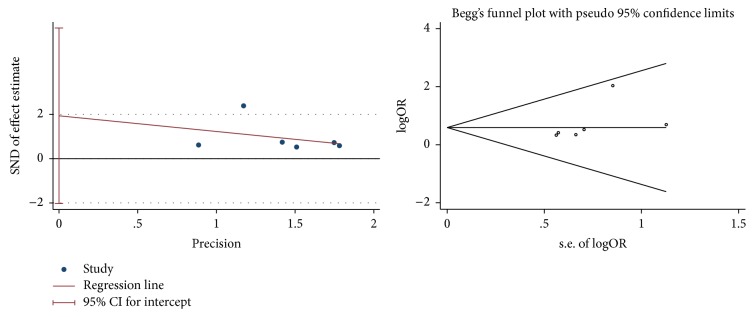
Egger's test and Begg's test of clinical effectiveness.

The funnel plot (Figure 8) was drawn by reviewer (Zhao-feng Shi) based on pooled odds ratio (OR) as the midpoint. The publication bias of clinical effectiveness in included studies was evaluated by comparing the symmetry of the funnel plot. The symmetry of funnel plot was assessed by two reviewers' cooperation (Juan Xie and Yi-quan Yan) in the visual aspect and both the reviewers considered that this image was symmetrical, which means the clinical effectiveness did not have the publication bias. We also conducted Egger's test and Begg's test to confirm whether the publication did exist. The calculating result of Egger's (*t* = 1.36, *P* = 0.247) and Begg's test (*z* = 1.88, *P* = 0.06) and images indicated that the publication bias did not exist [54] (Figure 9).

## 4. Discussion

### 4.1. Summary of the Main Evidence

This systematic review and meta-analysis have shown the clinical effectiveness of traditional Chinese medicine and related treatment and the amelioration of related evaluating criteria for atopic dermatitis (AD) therapy by collecting 24 high quality articles from 8 main international electronic databases. The evidence-based rules of traditional Chinese medicine and related treatment for AD have been conducted by this systematic review and meta-analysis. This study has four obvious advantages: (a) articles having good quality based on the Cochrane Collaboration's tools for the risk of bias assessment were included ensuring that the results trustworthy; (b) the articles' quality was evaluated by three reviewers' cooperation (Zhao-feng Shi, Juan Xie and Yi-quan Yan); (c) the subgroup analysis was well performed to find the source of heterogeneity; (d) the author was contacted by our reviewers if an appropriate study cannot be acquired as full-text; (e) the funnel plot, Egger's test, and Begg's test were conducted to evaluate the publication bias of articles' clinical effectiveness.

The result of the clinical effectiveness of traditional Chinese medicines and relevant treatments for atopic dermatitis (AD) therapy in meta-analysis showed that the experimental group (traditional Chinese medicine and relevant treatment) was irrelevant to the control group (placebo or western medicine). The subgroup analysis did not reveal the source of heterogeneity. The clinical evaluating criteria amelioration of SCORAD and SSRI for the traditional Chinese medicines and relevant treatments of AD provided the same conclusion as the clinical effectiveness. However, the result of EASI in meta-analysis illustrated that the experimental group (traditional Chinese medicine and relevant treatment) was superior to the control group (placebo or western medicine). Egger's and Begg's test and funnel plot did not reveal the publication bias for clinical effectiveness. In a word, under this circumstance, we could not give the firm conclusion on the effectiveness of traditional Chinese medicines and relevant treatments for AD except that we can include more relevant articles and credible results in the future meta-analysis.

### 4.2. The AD Adjunctive Therapy for Recommendations

Atopic dermatitis (AD) was recognized as a common inflammatory skin disease nowadays that bring much burden to humankind around the world, with the reported rate as high as 30% of children and 10% of adults [1]. Despite the obvious adverse effects (deranged metabolism, growth suppression, increased susceptibility to infection, and suppression of the hypothalamic-pituitary-adrenal axis) [55, 56], western medicines that included skin emollients, corticosteroids, antihistamines, and immunomodulation agents still have been put forward as AD standard therapy for a long time. Under the characteristics of a chronic course and repeated attack of AD, clinical physicians tried their best to find appropriate adjunctive treatments for better clinical control of AD symptoms. Traditional Chinese medicines and related treatments are popular examples of AD adjunctive therapy, which could be grouped in Complementary and Alternative Medical (CAM) treatments category.

In many Asian countries, such as China, Japan, South Korea, India, and Iran, traditional Chinese medicine (TCM) had been applied on clinical areas of AD for its outstanding efficacy and safety. TCM may have the ability to downregulate cells that associated with AD through antioxidant activities [57]. In addition to that, TCM may affect the structure of the developing dendritic cells (DCs) through losing their typical dendritic morphology and decreasing their expression of CD1a as well as the low affinity IgE receptor CD23 [58]. A China TCM Expert Consensus of AD was published in 2013, separating AD as four main syndromes based on theory of TCM: firstly, the brimming heat of heart and spleen syndrome, conducting the Chinese herbal prescription named* Sanxindaochiyin*; secondly, the heat of heart and spleen deficiency syndrome, conducting* Qinxinpeitufang*; thirdly, the spleen deficiency and dampness retention syndrome, performing* Xiaoerhuashitang*; fourthly, the syndrome of wind and dryness due to blood deficiency, conducting* Dangguiyinzi* [59]. However, there was still lack of modern randomized controlled trials to cautiously evaluate the TCM treatment of AD and the pharmacological mechanism of TCM for AD has been difficult to explain. Acupuncture and the relevant therapies (such as flying needle, auricular needle, and electroacupuncture) have been shown to be practical in patients with AD that may be due to the antipruritic effect of kappa-opioid receptor activated by high-frequency stimulation [60]. However, since many of them lack scientific verification, they should be performed under the supervision of physicians [12].

### 4.3. Limitation

This systematic review and meta-analysis had several potential limitations restricting the clinical application for AD: (a) Included studies are smaller than we expected although reviewers restricted the electric database numbers for high quality articles. (b) The number of participants in some included studies is small: Pfab. F only included 10 patients in their studies and Lee K.C.'s article only contains 15 patients [44, 47]. We consider that the limited participant numbers may be insufficient to evaluate the efficiency and safety of AD therapy. (c) The heterogeneity of included studies is obvious although reviewers conducted strictly study selection, data extraction, and quality analysis. And the subgroup analysis of studies also could not clearly reveal the source of heterogeneity. We estimate that the heterogeneity may come from the sample size, medicine application and dose, and publication years, as well as treating duration. We are required to understand that the random effects model was performed to pool data which cannot give exact and stable conclusion in this situation. (d) The traditional Chinese medicines and related treatments are diversified in the included articles: some studies used traditional Chinese herbal medicines and some studies performed acupuncture and related treatments. Also, therapy in control group could not reach a consensus as a uniform method.

## 5. Conclusion

In conclusion, this systematic review and meta-analysis did not provide the practical and beneficial results of traditional Chinese medicine and related treatment for atopic dermatitis (AD) therapy. Although the Eczema Area and Severity Index (EASI) amelioration in the meta-analysis showed a statistical difference, the clinical meaning was restricted by the shortage of included articles. So this aspect conclusion needs to be confirmed by more clinical studies in the future. As for evaluating aspects of clinical effectiveness, SCORAD amelioration, and SSRI amelioration, we did not find any firm outcome for the superiority of traditional Chinese medicine and related treatment in AD therapy. Further and deeper standard, multicenter, double-blind randomized clinical trials (RCTs) of traditional Chinese medicine and related treatment for AD were urgent to be conducted for more clinical evidences providing in the future. The underlying pharmacological mechanism of traditional Chinese medicine and related treatment needs to be researched and revealed for its future application of AD therapy.

## Figures and Tables

**Table 1:** General characteristics of included randomized controlled trials.

Author	Sample size		Age	Sex		Study design	Disease category	Diagnostic and evaluating criteria	Treating method	Treating duration	Dropout	Major outcome parameter	Side effect
	Treatment group	Control group		Treatment group	Control group								
Sheehan and Atherton 1992	47		1.5–18.1 years	Male: 27 Female: 20		Placebo-controlled, double-blind, crossover trial	Nonexudative atopic eczema	Simple scoring system	Traditional Chinese herbal formula (PSE101) versus placebo	5 months (assessed at 4 weekly intervals)	10	(1) Median clinical score for erythema and surface damage. (2) Clinical scores change for treatment group and control group. (3) The percentage changes of median and 95% CI median of clinical scores.	Eosinophilia and elevated serum IgE levels.

Fung et al. 1999	40		7–50 years	Male: 19 Female: 18		Double-blind, placebo-controlled, crossover study	Recalcitrant atopic dermatitis	The severity and extent of four clinical parameters (erythema, surface damage, lichenification, and scaling)	Traditional Chinese herbal formula (Zemaphyte) versus placebo	20 weeks (assessed at 4 weekly intervals)	3	(1) The median clinical scores variation for erythema, surface damage, lichenification, and scaling. (2) The comparison of median clinical scores at fourth week between the treatment group and control group. (3) The personal tendency of patients for Zemaphyte and placebo. (4) Complete blood picture and renal and liver function tests.	Hair loss, transient dizziness, gastrointestinal upsets, lichenoid eruption.

Lun and Rong 2000	25	25	15–60 years	Male: 31 Female: 19		Randomized controlled trial	Intractable cutaneous pruritus	—	Auricular acupuncture versus combination of cyproheptadine and calamine lotion	5 weeks	0	Therapeutic effects between two groups (curing, markedly effective, effective, ineffective).	No report.

Bai et al. 2007	120		15–60 years (mean age: 27.45 years)	Male: 42 Female: 78		Randomized controlled trial	Acute eczema	Clinical criteria of acute eczema comes from *Clinical Dermatology*	Traditional Chinese medicine method (Shuangfujin) versus normal saline group; boric acid group; Pikangwang group	4 days	0	(1) Clinical score variation of skin damage. (2) Clinical score variation of pruritus. (3) The total clinical effectiveness and basic recovery comparison. (4) Adverse effects. (5) Syndrome improvement. (6) Laboratory index.	Regional erythema eruption and burning sensation.

Hon et al. 2007	42	43	5–21 years	Male: 23 Female: 19	Male: 23 Female: 20	Randomized, double-blind, placebo-controlled study	Atopic dermatitis	SCORAD; CDLQI; allergic rhinitis score	Traditional Chinese herbal medicine concoction versus placebo	12 weeks	0	(1) Improvement from baseline in mean SCORAD. (2) Improvement from baseline in mean CDLQI scores. (3) Blood counts, eosinophil counts, total IgE levels, and liver and renal function.	Upper respiratory tract; infection; diarrhoea; abdominal pain; episodes of asthma.

Chen et al. 2008	38	37	18–54 years	Male: 3 Female: 35	Male: 5 Female: 32	Randomized controlled trial	Facial corticosteroid addictive dermatitis	Clinical criteria for facial corticosteroid addictive dermatitis and TCM syndrome differentiation criteria *(Xuexufengzao)*	Modified Wuhua decoction combined with levocetirizine versus levocetirizine	30 days	12	(1) Clinical scores. (2) The variation of symptom score before and after treatment. (3) The variation of skin erythema and TEWL before and after treatment. (4) Adverse effect.	Gastrointestinal upsets; mild drowsiness.

Senapati et al. 2008	25	25	Treatment group mean age: 16.09 years; control group mean age: 17.46 years	Male: 11 Female: 14	Male: 7 Female: 18	Randomized placebo-controlled trial	Atopic dermatitis	Hanifin and Rajka criteria: diagnostic features of atopic dermatitis; IISA	Evening primrose oil versus placebo	5 months	12	(1) The scores of study cases at baseline and at five monthly evaluations. (2) Effect of evening primrose oil versus placebo on total scores of patients of atopic dermatitis. (3) Adverse effect.	Not found.

Shi et al. 2008	25	22	Treatment group: 14–32 years; control group: 15–31 years	Male: 14 Female: 11	Male: 12 Female: 10	Randomized controlled trial	Atopic dermatitis	The UK working party's diagnostic criteria for atopic dermatitis derivation	Jiaweidangguiyin combined with loratadine versus loratadine	4 months	0	(1) The clinical effectiveness comparison between experimental group and control group. (2) The cytokine levels comparison before and after treating. (3) Safety and adverse effect.	Not found.

Xu et al. 2008	22	20	Treatment group: 52.09 ± 16.94 years; control group: 43 ± 16.53 years	Male: 10 Female: 12	Male: 10 Female: 10	Randomized controlled trial	Chronic eczema	Clinical criteria of chronic eczema comes from *Clinical Dermatology *and TCM syndrome differentiation criteria *(Xuexufengzao)*; SSRI and Eczema Area and Severity Index score	Herbal Saxifrage cream versus hydrocortisone	4 weeks	0	(1) Symptom score before treatment and SSRI after treatment in two groups. (2) Clinical efficacy in two groups.	No report.

Gao et al. 2009	30	30	Treatment group: 35.6 ± 13.8 years; control group: 37.5 ± 12.9 years	Male: 13 Female: 17	Male: 11 Female: 19	Randomized controlled trial	Chronic urticaria	Chronic urticaria comes from *the TCM disease diagnostic and effective criteria*	Penetrative needling of *Shendao *versus levocetirizine hydrochloride	12 weeks	0	(1) Comparison between acupuncture and medication groups in the symptom scores. (2) Comparison between acupuncture and medication groups in serum IgE level.	No report.

Kobayashi et al. 2010	37	40	20–40 years	—		A 6-month, Multicenter, double-blind, randomized, placebo-controlled study	Atopic dermatitis	The scoring system by the Atopic Dermatitis Severity Evaluation Committee of Japanese Dermatological Association	Traditional herbal medicine *(Hochu-ekki-to)* versus placebo	24 weeks	14	(1) Clinical efficacy of *Hochu-ekki-to*(time course change of skin severity score during examination; time course change of equivalent dosage of topical agent during examination). (2) Adverse effects and abnormal laboratory findings.	No report.

Pfab et al. 2010	30		18–50 years	Male: 16 Female: 14		A blinded, randomized, placebo-controlled, crossover trial	Atopic dermatitis	Visual analogue scale and the Eppendorf Itch Questionnaire	Acupuncture versus placebo	10 minutes	0	(1) Wheal and flare size. (2) Skin perfusion measured at the stimulus site. (3) The validated Eppendorf Itch Questionnaire.	No report.

Cheng et al. 2011	47	24	Treatment group mean age: 12.2 years; control group mean age: 13.6 years	Male: 25 Female: 22	Male: 12 Female: 12	A randomized, double-blind, placebo-controlled trial	Refractory atopic dermatitis	Hanifin and Rajka criteria: diagnostic features of atopic dermatitis	Chinese herbal product (Xiao-Feng-San) versus placebo	8 weeks	10	(1) Improvement in scores (clinical lesion; erythema; surface damage; pruritus; sleep) between baseline and week 8. (2) Score improvement (clinical lesion; erythema; surface damage; pruritus; sleep) from baseline to weeks 4, 8, and 12. (3) Immunologic markers variation before and after treatment. (4) Laboratory index.	Transient elevation of aspartate aminotransferase; gastrointestinal upsets.

Hon et al. 2012	42	43	5–21 years	—		Randomized, double-blind, placebo-controlled study	Atopic dermatitis	Hanifin and Rajka criteria: diagnostic features of atopic dermatitis; SCORAD; allergic rhinitis scores; the Children's Dermatology Life Quality Index	Traditional Chinese herbal medicine versus placebo	12 weeks	0	(1) Percentage improvement of SCORAD from baseline. (2) The total allergic rhinitis scores between two groups. (3) Adverse effects.	Not found.

Pfab et al. 2011	5	5	Mean age: 25.2 ± 4.5	Male: 8; female: 2		Unicenter, single-blinded, prospective, randomized clinical pilot trial	Atopic eczema	Visual analogue scale; SCORAD	Acupuncture versus blank group	33 days	0	(1) SCORAD comparison between two groups. (2) Itch intensity (VAS) between two groups. (3) Basophil activation test comparison between two groups.	No report.

Xiu and Wang 2011	100	100	Treatment group: 14 months to 60 years; control group: 2 to 65 years	Male: 60 Female: 40	Male: 55 Female: 45	Randomized controlled trial	Chronic urticaria	—	Acupoint injection with autoblood versus medicine group	30 days	0	(1) Clinical effectiveness comparison between two groups. (2) The mean onset time between two groups. (3) The cured numbers for different kinds of chronic urticaria.	No report.

Choi et al. 2012	12	12	Treatment group: 18.6 ± 8.4 years; control group: 14.2 ± 6.0 years old	Male: 5 Female: 7	Male: 5 Female: 7	Parallel, randomized, active-controlled, double-blind trial	Atopic dermatitis	Hanifin and Rajka criteria: diagnostic features of atopic dermatitis and dampness-heat pattern type of AD; SCORAD and EASI score	TJ-15 combined with TJ-17 versus TJ-15	4 weeks	5	(1) Change of SCORAD score. (2) Change of EASI score. (3) Clinical safety evaluated by laboratory index.	No report.

Lee et al. 2012	8	7	19–79 years; treatment group median age: 34 years; control group median age: 36 years	Male: 4 Female: 4	Male: 6 Female: 1	Single-center, randomized controlled trial	Atopic dermatitis	VAS scores, EASI scores, and IGA	Acupressure versus using any prescription or over-the-counter medications or lotions (except acupressure technique)	4 weeks	0	(1) Change in VAS between control and experimental groups. (2) Change in IGA score between control and experimental groups. (3) Change in EASI score between control and experimental groups.	No report.

Pfab et al. 2012	20		Mean age: 23.3 ± 1.7 years	Male: 6 Female: 14		A patient- and examiner-blinded, randomized, placebo-controlled, crossover trial	Atopic dermatitis	Atopic dermatitis diagnosis [scoring atopic dermatitis (SCORAD) > 20]; VAS; EIQ	VAp, VAa, VC versus PAp, PAa, and PC	20 minutes	0	(1) Mean itch intensity. (2) Wheal and flare size. (3) Qualitative assessment of itch intensity (EIQ). (4) Mean attention scores. (5) Evaluation of blinding.	No report.

Wu et al. 2012	50	35	18–70 years	Male: 28 Female: 22	Male: 15 Female: 20	Randomized controlled trial	Atopic dermatitis	Clinical criteria of chronic urticaria comes from *Clinical Dermatology*	Yiqi Huoxue Qufeng decoction versus Fuyang granule	8 weeks	0	(1) The clinical effects comparison between two groups. (2) The DAO comparison between two groups. (3) The positive rate of IgE comparison between two groups.	No report.

Quan et al. 2014	30	30	Treatment group: 24.3 years; control group: 23.45 years	Male: 13 Female: 16	Male: 14 Female: 16	Randomized controlled trial	Atopic dermatitis	The western medicine diagnostic criteria and traditional Chinese medicine diagnosis standard; SCORAD scores	Flying needle combined with herbal medicine versus herbal medicine combined with regular skin-care	3 months	1	(1) The comparison of SCORAD scores. (2) Total effective rate. (3) Side effect.	No report.

Liu et al. 2015	250		5–25 years	Male: 107 Female: 143		Randomized controlled trial	Atopic dermatitis	The criteria of Hanifin and Rajka; SCORAD; QoL scores; self-assessment scores	PTQXT versus combination TCM therapy versus control group (mometasone furoate)	12 weeks	0	(1) Efficacy outcomes (SCORAD, QoL scores, self-assessment scores). (2) Adverse effects. (3) Test of blinding.	Slight diarrhoea.

Mehrbani et al. 2015	24	18	Treatment group: 28.62 ± 2.30 years; control group: 24.33 ± 1.50 years	Male: 4 Female: 20	Male: 2 Female: 16	A randomized, double-blind, placebo-controlled clinical trial	Moderate-to-severe atopic dermatitis	The criteria of Hanifin and Rajka	Dodder seed extract versus placebo	15 days	10	(1) Efficacy. (2) Safety profile.	Anorexia; mild gastrointestinal problems.

Yen and Hsieh 2016	16	17	Treatment group: 32.9 ± 13.4 years; control group: 39.6 ± 12.3 years	Male: 10 Female: 5	Male: 10 Female: 6	A preliminary, randomized, controlled, open-label study	Atopic dermatitis/ eczema	The criteria of Hanifin and Rajka; EASI scores and TIS scores	TYO (Tzu-Yun ointment) versus TS cream (topical steroid)	8 weeks	0	(1) Effect of TYO and TS cream on EASI and TIS scores in patients with eczema/atopic dermatitis. (2) Comparison between effects of TYO and TS cream on EASI and TIS scores in patients with eczema/atopic dermatitis.	No report.

*Note*. SCORAD: SCORing Atopic Dermatitis; CDLQI: Children's Dermatology Life Quality Index; IISA: Intensity Item Score Aggregate; TCM: traditional Chinese medicine; SSRI: Symptom Score Reducing Index; EASI: Eczema Area and Severity Index; VAS: visual analogue scale; IGA: Investigator's Global Assessment; EIQ: Eppendorf Itch Questionnaire; QoL: Quality of Life; PTQXT: Pei Tu Qing Xin Tang; TIS: Three-Item Severity.

**Table 2:** The risk bias evaluation of articles.

Article names	Random collection method	Allocation concealment	The blinding method	Outcome data integrity	The outcome data of selective report	No other bias sources	The level of bias risk
Sheehan and Atherton 1992	Y	Y	Y	Y	Y	U	Low
Fung et al. 1999	Y	Y	Y	Y	Y	U	Low
Lun and Rong 2000	Y	U	U	Y	Y	U	Low
Bai et al. 2007	Y	U	U	Y	Y	U	Low
Hon et al. 2007	Y	Y	Y	Y	Y	U	Low
Chen et al. 2008	Y	U	U	Y	Y	U	Low
Senapati et al. 2008	Y	Y	U	Y	Y	U	Low
Shi et al. 2008	Y	U	U	Y	Y	U	Low
Xu et al. 2008	Y	U	U	Y	Y	U	Low
Gao et al. 2009	Y	U	U	Y	N	U	High
Kobayashi et al. 2010	Y	Y	Y	Y	Y	U	Low
Pfab et al. 2010	Y	Y	Y	Y	Y	U	Low
Cheng et al. 2011	Y	U	U	Y	Y	U	Low
Hon et al. 2012	Y	Y	Y	Y	Y	U	Low
Pfab et al. 2011	Y	Y	Y	Y	Y	U	Low
Xiu and Wang 2011	Y	U	U	Y	Y	U	Low
Choi et al. 2012	Y	Y	Y	Y	Y	U	Low
Lee et al. 2012	Y	Y	U	Y	Y	N	Low
Pfab et al. 2012	Y	Y	Y	Y	Y	U	Low
Wu et al. 2012	Y	U	U	Y	Y	U	Low
Quan et al. 2014	Y	U	U	Y	Y	U	Low
Liu et al. 2015	Y	Y	Y	Y	Y	U	Low
Mehrbani et al. 2015	Y	Y	Y	Y	Y	U	Low
Yen and Hsieh 2016	Y	U	U	Y	Y	U	Low

*Note*. Y refers to yes, N refers to no, and U refers to unknown.
